# Species Identification of Archaeological Skin Objects from Danish Bogs: Comparison between Mass Spectrometry-Based Peptide Sequencing and Microscopy-Based Methods

**DOI:** 10.1371/journal.pone.0106875

**Published:** 2014-09-26

**Authors:** Luise Ørsted Brandt, Anne Lisbeth Schmidt, Ulla Mannering, Mathilde Sarret, Christian D. Kelstrup, Jesper V. Olsen, Enrico Cappellini

**Affiliations:** 1 Centre for Textile Research, University of Copenhagen, Copenhagen, Denmark; 2 Centre for GeoGenetics, University of Copenhagen, Copenhagen, Denmark; 3 Department of Environmental Archaeology and Materials Science, The National Museum of Denmark, Copenhagen, Denmark; 4 Department of Ancient Cultures in Denmark and the Mediterranean, The National Museum of Denmark, Copenhagen, Denmark; 5 European School of Chemistry, Polymers and Materials Science, University of Strasbourg, Strasbourg, France; 6 Novo Nordisk Foundation Center for Protein Research, University of Copenhagen, Copenhagen, Denmark; New York State Museum, United States of America

## Abstract

Denmark has an extraordinarily large and well-preserved collection of archaeological skin garments found in peat bogs, dated to approximately 920 BC – AD 775. These objects provide not only the possibility to study prehistoric skin costume and technologies, but also to investigate the animal species used for the production of skin garments. Until recently, species identification of archaeological skin was primarily performed by light and scanning electron microscopy or the analysis of ancient DNA. However, the efficacy of these methods can be limited due to the harsh, mostly acidic environment of peat bogs leading to morphological and molecular degradation within the samples. We compared species assignment results of twelve archaeological skin samples from Danish bogs using Mass Spectrometry (MS)-based peptide sequencing, against results obtained using light and scanning electron microscopy. While it was difficult to obtain reliable results using microscopy, MS enabled the identification of several species-diagnostic peptides, mostly from collagen and keratins, allowing confident species discrimination even among taxonomically close organisms, such as sheep and goat. Unlike previous MS-based methods, mostly relying on peptide fingerprinting, the shotgun sequencing approach we describe aims to identify the complete extracted ancient proteome, without preselected specific targets. As an example, we report the identification, in one of the samples, of two peptides uniquely assigned to bovine foetal haemoglobin, indicating the production of skin from a calf slaughtered within the first months of its life. We conclude that MS-based peptide sequencing is a reliable method for species identification of samples from bogs. The mass spectrometry proteomics data were deposited in the ProteomeXchange Consortium with the dataset identifier PXD001029.

## Introduction

### BACKGROUND

Skin and leather artefacts are rarely found in archaeological contexts, as biogenic and non-biogenic factors rapidly cause their complete decomposition [1]. Nevertheless, archaeological objects that derive from animal soft tissues, such as skin and leather, may survive in environments with exceptional conditions, such as anoxia, waterlogging, low temperature, high salt concentration, or extreme dryness [2], [3]. One favourable environment in this regard is the raised bogs of North Western Europe, as their acidic and anaerobic soil with low average temperature and content of sphagnan inhibit microorganism proliferation and promote skin, hair, and other soft tissue preservation by natural tanning processes [4]. Therefore, a significant number of deposited ancient textiles and skin garments had been preserved in raised bogs and unearthed during peat cutting [5]–[7].

Danish peat bogs, in particular, have yielded one of the world’s finest collections of prehistoric textiles and skins, including more than 68 well-preserved prehistoric skin objects [8], [9] dating from 920 BC to AD 775 approximately, i.e. the Danish Late Bronze and Iron Ages [10], [11]. The skin object collection predominantly consists of capes and shoes, and while some retain nearly full hair content, others lack parts, or all of the original hair. The 24 skin capes, found either singly or associated with male or female bog bodies, are considered to represent unisex clothing [5]. The capes were sewn together of 4–7 large polygons or rectangular elements, each representing an entire animal skin, and several smaller pieces of skin. The majority of the capes are symmetrically designed, whereas a minor part displays an asymmetric design (Fig. S1 in File S1). The largest skin elements measure up to approximately 90 cm in height, but on average they measure between approximately 30–50 cm in height, and 25–40 cm in width. An essential feature noted on some of the skin elements is the dorsal line of hair often placed in the centre of the elements, indicating that skins were cut symmetrically along the spine of the animal, which today, too, is the customary manner of cutting an animal skin.

These exceptional finds provide a unique opportunity to not only investigate prehistoric skin costume technologies, but also understand which animal species were used in the process. This is crucial as species-specific morphological characteristics of skins, such as size, thickness, flexibility and function determine costume properties and the number of elements required to produce a skin object [12]–[14]. The types of skin utilized also define the pertinent manufacturing techniques and possible product types. Moreover, species identification of archaeological skins can enhance our understanding of prehistoric animal husbandry. This includes the exploitation and preferences of animal products as meat, milk, wool and skins, and the management strategies of flocks required to produce these products.

### PREVIOUS METHODS FOR SPECIES IDENTIFICATION OF ARCHAEOLOGICAL SKIN OBJECTS

Attempts to identify the species origin of archaeological skin objects have been carried out since the 19^th^ century [15]. Until recently, skins with a preserved pelage were primarily subjected to identification via either macroscopic inspection, or by using light and electronic microscopy to investigate the hair morphology [9], [16]–[20]. This method is also extensively applied in forensic science [21], [22]. The distribution of primary and secondary hairs is characteristic for each animal species, and the position and size of the various hairs produce a species-specific surface pattern of the grain or the dermal papillary layer, that varies over the body. The recognition of this so-called “grain pattern” is a further feature that can be used for the identification of animal species [14]. Grain pattern is primarily used on de-haired skin or fur skin with lost pelage. The recognition of the cross-section of the dermal layer by means of light microscopy can also be employed as a tool for the identification of animal species [14], [23], however as skin sampling was restricted, this analysis was not included in this work.

Species identification is also commonly performed utilizing hair through the evaluation of ‘diagnostic’ morphological traits, including: hair diameter, length of the fibre, shape and distance of the cuticles scales, appearance and dimension of the medulla and cortex [24], [25], and cross-sectional shape. These traits are evaluated and identified by comparison to atlases and reference collections [16], [17], [25]–[29]. Thus far, the majority of the skins of the Danish capes have been identified by microscopy as domesticated animals, such as sheep, goat and cattle. Otter (*Lutra lutra*) and wolf (*Canis lupus*) skins were, however, also identified in one cape, and deerskin (*Cervus*) in another [8]. Despite being widely applied, the reliability of species identification based on the light and electron microscopic observation of skin and hair morphology is subject to intense debate [16], [17], [24], [25], [30], [31]. A primary matter of concern is that the reproducibility of the method requires extended knowledge and experience. Furthermore, hair morphology can diverge within the same species, between different parts of the animal’s surface, or according to age, sex, seasonality, nutrition and health. These challenges are further complicated in archaeological contexts. First, fiber atlases are based on modern species and at present there is no fiber atlas available that includes archaeological material. This is problematic, as domestication and selective breeding of animals have altered hair morphology, which is reflected in the appearances of the scale structure and medulla [17], [32], [33]. Secondly, archaeological hairs are often poorly preserved [34] and the degradation of prehistoric hairs can transform the appearance of the scales and medulla, which complicates the identifications [35]–[37]. Thirdly, environmental conditions can lead to the preservation of only partial fibres. These can yield misleading identifications, as scales and types of primary follicles differ, to some extent, between areas of the hair. Overall, it is evident that species identification based on the microscopic analysis of ancient hairs is not straightforward, thus rendering it desirable to develop alternative, ideally more reliable, approaches for the species identification of skins.

In recent decades, new methods based on the analyses of ancient biomolecules have been applied for the species identification of hide and leather. An ancient DNA-based approach was successfully applied to ancient parchment, bookbinding and clothing of hide and leather [38], [39]. The success of DNA-based approaches, however, depends on DNA preservation, which is conditioned by the diagenetic conditions that the sample experienced during archaeological deposition. The acidity and generally high amounts of molecules identified as PCR inhibitors in peat bogs affect aDNA preservation and strongly hampers its potential for amplification by PCR [8], [40], [41]. This is equally the case for skins and textiles that have been subject to tanning or mordanting processes [39], [42].

More recently, an alternative molecular approach for species identification, adopting mass spectrometry (MS) to analyse collagen and keratin residues extracted from small archaeological bone fragments, as well as skin and fur, was presented [43]–[51]. Collagen preservation levels in ancient skin objects, associated with highly hierarchical structural constraints and macroscopic protein quantities, suggest that, MS-based ancient peptide sequencing is applicable to samples from bogs, despite their exposure to harsh diagenetic conditions. Recently, methodological improvements and protocol optimisation, taking ancient protein characteristics into account, have enabled the identification of considerably more proteins than achievable hitherto [52]–[54]. Moreover, protein analysis holds the advantage of not being based on enzymatic amplification and consequently not being affected by conventional PCR inhibitors, overcoming the limits of aDNA analysis from ancient recalcitrant contexts [8].

We explored the potential of MS-based high throughput ancient peptide sequencing as a reliable approach for the species identification of archaeological skin objects from peat bogs. Unlike previous methods based on mass fingerprinting of peptides from selected collagen and keratin molecules, the shotgun sequencing approach aims to identify the total extracted ancient proteome, with no specific target selected in advance. In this study, we subjected samples from eleven archaeological skin objects to species identification employing three different approaches. Two of these rely on microscopy: the first combines macroscopic observation (MO) of the skin and inspection of the associated hairs by light microscopy (LM), while the second adopts light and scanning electron microscopy (SEM)-based observation of the hair morphology. The third approach is based on ancient peptide sequencing by MS. The conclusions reached by the three methods are compared, and the advantages and limitations of the various approaches discussed.

## Materials

**Figure 1:**
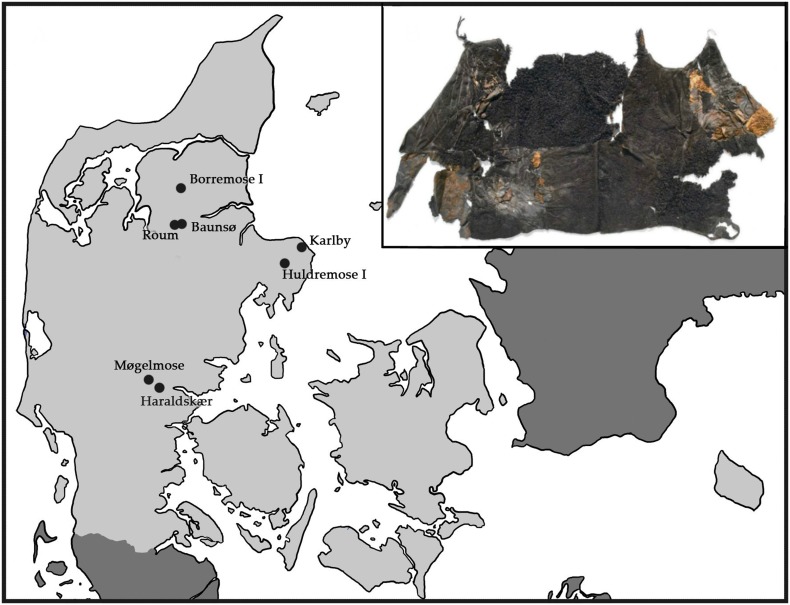
Locations where the investigated archaeological skin objects were found. Appearance of the skin cape from Huldremose I (inset). Photo by Roberto Fortuna, the National Museum of Denmark.

Twelve samples from eleven skin garments (ten capes and a tunic) from seven peat bog localities in Denmark were selected for this study (Fig. 1, Table 1, Fig. S1 in File S1). All samples derive from the collection of skin objects at the National Museum of Denmark. The dataset for each garment (except for the Huldremose I find, for which two samples were collected from two different skin elements) consisted of three samples extracted from the same skin element, as these sewn together skin elements may derive from different species. A skin sample, measuring approximately 2×2 mm, was cut off for MS-based peptide sequencing, together with a few hairs for microscopy analyses (Fig. S1 in File S1). To validate the MS approach, three modern reference samples were also analysed (Table 1), representing the three common domesticated species that the archaeological samples most likely derived from (cow, goat, sheep). The references were sampled from two historic skin samples from the Natural History Museum of Denmark, known to derive from domestic sheep and goats, and from a cattle skin provided by a local slaughterhouse.

**Table 1:** Archaeological skin objects from Danish peat bogs, and modern control samples investigated.

Sample no.	Locality	Museum no.	Calibrated 14C date BP	Dating
			(95, 4% probability)	
**1**	Baunsø*	NM D11103a	AD 20–220	
			Dating by cape b (Ua-33586)	
**2**	Baunsø	NM D11103b	AD 20–220	Ua-33586
**3**	Baunsø	NM D11103c	AD 20–220	-
			Dating by cape b (Ua-33586)	
**4**	Borremose I*	NM C26450	365–116 BC	
			Dating by textile (AAR-11678)	
**5**	Huldremose dark*	NM C3471	350–41BC	-
			Dating by textile (AAR-11675)	
**6**	Huldremose light*	NM C3471	350–41BC	-
			Dating by textile (AAR-11675)	
**7**	Karlby	NM D4854b	200 BC–AD 90, 170 BC–AD 140	-
			Dating by textile (Ua-3998, Ua-3999)	
**8**	Karlby	NM D4854c	200 BC–AD 90, 170 BC–AD 140	-
			Dating by textile (Ua-3998, Ua-3999)	
**9**	Karlby	NM D4854e	200 BC–AD 90, 170 BC–AD 140	-
			Dating by textile (Ua-3998, Ua-3999)	
**10**	Møgelmose*	NM 16316	520–150 BC, AD 1–550**	OxA-1188, Ua-334
**11**	Roum	NM C37412	50 BC–AD 80	Ua-33584
**12**	Haraldskær*	NM 3705	508–211 BC	AAR-11659
	Domestic sheep	CN3213	Dating not performed	Sampled in 1959
	(*Ovis aries*)			
	Domestic goat	CN3196	Dating not performed	Sampled in 1959
	(*Capra hircus*)			
	Domestic cattle	-	Dating not performed	Sampled in 2012
	(*Bos taurus*)			

The archaeological skin objects date to the Pre-Roman Iron Age: 500–1 BC, Early Roman Iron Age: AD 1–200, and Late Roman Iron Age: AD 200–400 [10].

* Samples prepared following procedure A during MS-peptide sequencing analysis.

** The youngest dating is the most probable according to Ebbesen [11].

### Ethics Statement

The archaeological samples (1–12, Table 1) were provided by the National Museum of Denmark, Frederiksholms Kanal 12, DK-1220 Copenhagen K. The historical samples (CN3213 and CN3196) were obtained from the Natural History Museum of Denmark, Zoological Museum, Universitetsparken 15, DK-2100 Copenhagen Ø. All necessary permits were acquired for the described study, which complied with all relevant regulations. The modern cattle sample was obtained with the kind permission of Lennart Engberg Carlsen from the slaughterhouse Anubis, Department of Basic Animal and Veterinary Sciences, Grønnegårdsvej 7, DK-1870 Frederiksberg.

### Data deposition note

The mass spectrometry proteomics data were deposited in the ProteomeXchange Consortium (http://proteomecentral.proteomexchange.org) via the PRIDE partner repository [55] with the data set identifier PXD001029.

## Methods

Three different methods were applied to the same skin elements. Microscopy-based method 1 “MO+LM” was performed by Anne Lisbeth Schmidt, at the National Museum of Denmark’s Conservation Department, microscopy-based method 2 “LM+SEM” by Antoinette Rast-Eicher, at ArchaeoTex, Switzerland, and MS-based ancient peptide sequencing method 3 “MS” was performed at the Centre for GeoGenetics by Luise Ørsted Brandt and Enrico Cappellini.

### Species identification by microscopy and macroscopic observation

Two microscopy methods were applied for the purpose of traditional species identification. Both species identification methods used light microscopy: the first method 1, “MO+LM”, combined light microscopy with macroscopic observation of the skins elements, whereas the second method, “LM+SEM”, combined light microscopy with SEM.

Transmitted light microscopy focused on the observation of primary and secondary hair [16], [29], [56], [57]. Cross-sectional photos were taken with an Axio Scan.Z1 Slide Scanner from Carl Zeiss Microscopy. Species identification was based on scale pattern and absence/presence and shape of medulla, according to the terminology of Wildman [25], and the shape of cross-sectioned hair, according to Teerink [16]. As reference, a range of fibre atlases was used [16], [25]–[29], in combination with modern mammalian hair samples, which were kindly lent by the Natural History Museum of Denmark. In the present study the grain pattern was investigated for the sole de-haired sample 12 (Haraldskær NM3705).

Macroscopic observations of skin size, thickness and flexibility, as well as the general appearance of the hair in the pelage, were also applied in “MO+LM” to support the species identifications [8]. The appearance comprises hair length, shape, the presence or absence of hair curls, primary and secondary hair and dorsal hair stripes.

SEM analysis was restricted to hair samples [58], [59], through comparison against several atlases and a private collection of reference samples [17], [30], [60] (Fig. S2 in File S1), in combination with an initial identification by light microscopy. The primary criteria for hair micromorphology-based identification of the commonest domesticated species (sheep, goat, cattle and horse) following Meyer et al. [17] are listed in Table S1 in File S1.

### Species identification by MS-based ancient peptide sequencing

The third method used mass spectrometry to sequence ancient protein residues. The samples were analysed in two distinct batches adopting different sample preparation approaches. Conditions adopted for liquid chromatography-electrospray ionisation (LC-ESI) and high-resolution tandem mass spectrometry (MS/MS) are described in details as Supplementary Information (see Text S1 in File S1) and referred to as procedure ‘A’ and ‘B’. Samples marked with an ‘*’ symbol in Table 1 were prepared following procedure ‘A’, while all the other samples were prepared following procedure ‘B’.

## Results

Species identification results were fully compatible in all three methods for six of the twelve samples (Table 2). In the remaining six cases, while the microscopy-based methods consistently disagree with each other, the MS-based peptide sequencing agrees with one of the two microscopy-based methods in four out of six cases. The three methods generally agree on the identifications of sheep (sample 3–7, 11) except for sample 9, in this case “LM+SEM” suggests a discordant identification. For the identification of other species, consensus seems harder to reach. In one case (sample 1), the three methods reached three different conclusions. In the case of sample 12, “MO+LM” and “MS” reached different results, whereas “LM+SEM” was not applicable as hair for only one microscopic analysis was available. In cases where “LM+SEM” and “MS” identified cattle, “MO+LM” identified goatskin (sample 2 and 10). In two cases “LM+SEM” and “MS” disagree between horse and goat identifications (sample 1 and 8).

**Table 2:** Species identification of the archaeological skin samples, based on the three methods applied.

Sample no.	Find	MO+LM	LM+SEM	MS	MO+LM vsLM+SEM	MO+LM vsMS	LM+SEM vsMS
**1**	Baunsø, NM D11103a	Cattle	Horse	Goat	≠	≠	≠
**2**	Baunsø, NM D11103b	Goat	Cattle	Cattle	≠	≠	=
**3**	Baunsø, NM D11103c	Sheep	Sheep	Sheep	=	=	=
**4**	Borremose I, NM C26450	Sheep	Sheep	Sheep	=	=	=
**5**	Huldremose I dark, NM C3471	Sheep	Sheep	Sheep	=	=	=
**6**	Huldremose I light, NM C3471	Sheep	Sheep	Sheep	=	=	=
**7**	Karlby, NM D4854b	Sheep	Sheep	Sheep	=	=	=
**8**	Karlby, NM D4854c	Goat	Horse	Goat	≠	=	≠
**9**	Karlby, NM D4854e	Sheep	Cattle	Sheep	≠	=	≠
**10**	Møgelmose, NM 16316	Goat	Cattle	Cattle	≠	≠	=
**11**	Roum, NM C37412	Sheep	Sheep	Sheep/goat	=	=	=
**12**	Haraldskær, NM 3705	Cattle	*	Goat		≠	

“MO+LM”: macroscopical observation and light microscopy, “LM+SEM”: light microscopy and scanning electron microscopy, “MS”: Mass Spectrometry-based peptide sequencing. *This item is thought to be deliberately de-haired and only few hairs are preserved on the surface. Therefore there was only sufficient hair for one microscopic analysis.  = /≠ indicate same/different species identification achieved by the methods compared.

The sample preparation procedure used for MS-based ancient peptide sequencing yielded protein recoveries estimated in the range between 1.32 and 20.13 mg of protein/g of extracted skin (Table S2 in File S1). While yields for proteins extracted from ancient skins have not been reported earlier, these values appear to be similar or superior to the approximately 5 mg protein/g bone obtained from ancient bone [52]. Skin samples from the same localities present similar values, suggesting that the protein yield could be related to archaeological site-specific preservation conditions. Statistics, reporting numbers of identified proteins and peptides for each sample, as well as the relative supporting tandem MS spectra, indicate that sample preparations based on procedure “A” enabled the recovery of richer datasets (Table S2 in File S1). Most of the proteins identified are collagens and keratins, in agreement with the nature of the samples analysed. However, the adopted approach also allowed the identification of proteins and peptides not previously reported in ancient skin samples [43], such as, leucine-rich-containing protein, serum albumin, selenium-binding protein and haemoglobin foetal subunit beta (Tables S3 in File S1).

**Figure 2:**
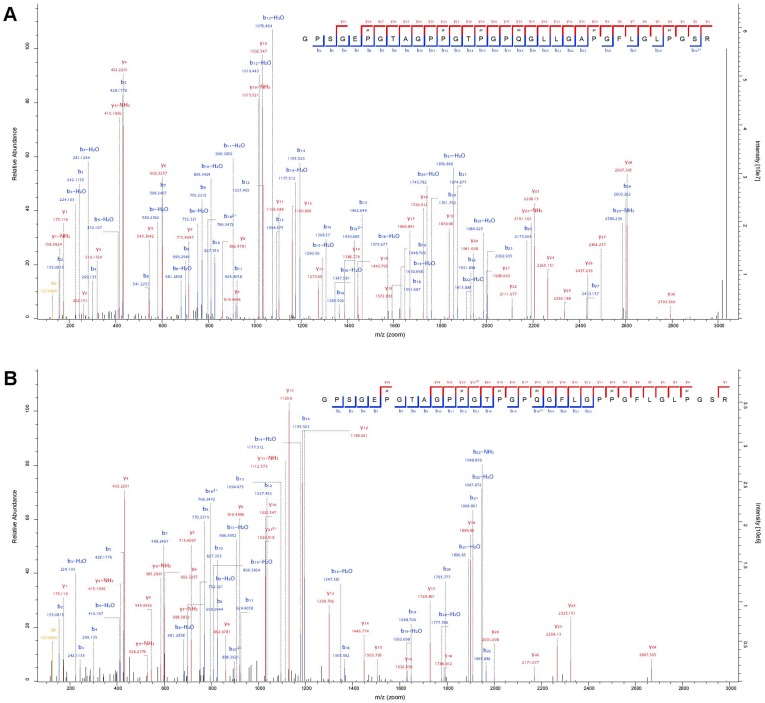
Examples of tandem MS spectra supporting identification of the type I alpha-2 collagen (COL1A2) sheep/goat diagnostic peptide [43], [62]. A) MS/MS spectrum from the sample 9, from Karlby (D4854e), confidently assigned to amino acid sequence GPSGEPGTAGPPGTPGPQGLLGAPGFLGLPGSR, diagnostic for sheep. B) MS/MS spectrum from sample 8, (Karlby D4854c), confidently assigned to amino acid sequence GPSGEPGTAGPPGTPGPQGFLGPPGFLGLPGSR, diagnostic for goat.

The search strategy adopted enabled the determination of a set of species-specific peptides (Tables S3, S4 and S5 in File S1), within publicly available protein databases. Based on spectra matched against the complete bovine reference protein list and extended lists of sheep and goat proteins available in NCBI RefSeq (http://www.ncbi.nlm.nih.gov/refseq), it was possible to identify at least one species-diagnostic peptide for all samples except two: 9 and 11. Peptides were considered diagnostic when, after BLAST search [61] against the entire nrNCBI protein database, they were assigned to a single species, or to a limited number of species among which only one can be considered plausible, based on the nature of the samples, such as the size of the skin element, or their geographic origin. For example, peptides equally present in cattle (*Bos taurus*), water buffalo (*Bubalus bubalis*) and yak (*Bos mutus*) were considered diagnostic for cattle. For samples lacking at least one species-diagnostic peptide, i.e. sample 9 and 11, species identification was attempted based on a set of peptides [43], [62], only compatible with one species (Fig. 2 and Table S5 and S6 in File S1).

Two skin samples were identified as bovine (sample 2 and 10), six as sheep (*Ovis aries*, sample 3–7 and 9), and three as goat (*Capra hircus,* sample 1, 8, 12), while for one sample (sample 11), identified as ovine, it was not possible to detect any marker to discriminate between sheep and goatskin (Table 2). The preparation of modern comparable material from known species with the same procedure, enabled the recovery of a higher number of diagnostic peptides (Tables S4 and S5 in File S1) for each sample. This is in full agreement with the recent origin of the material and its storage in favourable conditions. Only a limited number of the species-specific peptides identified (Fig. 2 and Table S5 in File S1), were previously reported in literature describing ancient samples [43], [46]–[49], [63].

**Figure 3:**
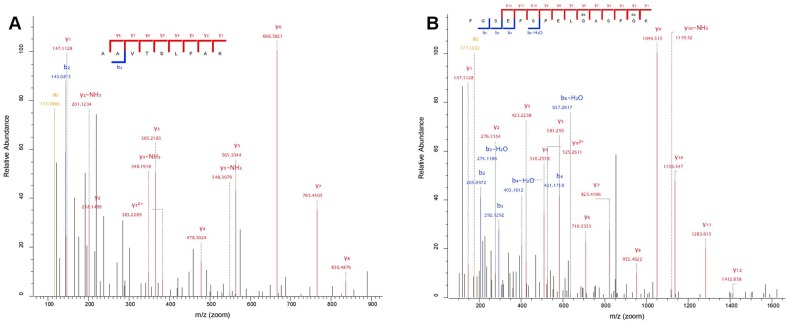
Tandem MS spectra from sample 10, Møgelmose supporting identification of bovine fetal hemoglobin subunit beta (UniProt accession number: P02081). MS/MS spectra confidently identified two peptide sequences: A) AAVTSLFAK and B) FGSEFSPELQASFQK.

The MS-based approach recovers additional information of particular interest for archaeological reconstruction and the understanding of the exploitation of natural resources in antiquity. An example is the secure identification of peptides uniquely assigned to bovine haemoglobin foetal subunit beta (UniProt accession number: P02081) in sample 10 (Fig. 3 and Table S3 in File S1). This protein is expressed in the foetus during the final months of pre-birth development and in those immediately after. At birth it represents approximately 40 to 100% of the total haemoglobin in a calf, and its concentration then diminishes rapidly until completely replaced by adult haemoglobins on average approximately two to three months after birth [64]. The identification of a protein expressed in such a defined time frame during pre- and immediately post-natal calf development allows a precise pinpointing of the time at which the animal was slaughtered for garment production. Although bovine haemoglobin is usually listed as a common proteomics contaminant, the absence of haemoglobin foetal subunit beta-specific peptides (reported in Fig. 3 and Table S3 in File S1) in all the other samples analysed in the same batch and in negative controls strongly suggests that these peptides were genuinely recovered from the archaeological sample and not indicators of a contamination. At present and to the best of our knowledge, there is no other approach that can provide this type of information for archaeological skin samples.

## Discussion

The lack of consensus among the results of the microscopy-based methods, for half of the analysed samples, illustrates that their use as a tool in species identification is not straightforward. Most likely, the challenges hampering the macroscopic and microscopic identification of archaeological skins and hair constitute part of the explanation for these discrepancies. However, the two microscopic methods applied hold different advantages for species identification. Light microscopy provides information on the colour or pigmentation of the hair and the structure of the medulla, while SEM allows enhanced observation of the scale patterns due to high magnification and a 3D view. The macroscopic observation of the size and thickness of skin elements immediately excludes several species from further consideration. For instance, sample 1 was previously identified as deer [65] and during this study it was assigned to three different species: cattle, horse, and goat. The species identification of this skin therefore seems particularly difficult. The size of the skin element, 70 cm in length from neck to tail, is compatible with cattle and traditional Danish goat breeds [66], while its thickness and hair length leads to its identification as cattle skin. However, the absence of an accurately identified archaeological skin reference material cannot completely exclude ambiguous conclusions based on the observation of these traits.

The result of “LM+SEM” suggested that the skin in question was horse skin (Fig. S3). However, distinguishing between horse and goatskin with light microscopy and SEM is difficult as illustrated in Table S1 and Fig. S4 in File S1. MS-based identification of sample 1 as goatskin, relies on several diagnostic peptides (Fig. S5 and Table S5 in File S1). The relevance of some of these peptides as a species marker has already been reported [63]. Based on the peptide signal, and the difficulties in microscopic identifications, the MS-based identification of the sample as goatskin is considered to be conclusive.

In addition, the availability of the cattle reference proteome and the recent public release of extended lists of sheep and goat proteins allowed for an exhaustive peptide and protein identification without limiting the search to a subset of the most abundant collagens and keratins [43], [62]. Although publicly available protein lists currently used to assign peptide sequences to spectra are only complete, or significantly extended, for a relatively few mammal species, the number of mammal proteomes extensively covered is rapidly increasing. During the preparatory stage of our work, we observed, and took advantage of, the inclusion of extended protein lists for both sheep (*O. aries*) and goat (*C. hircus*) in public databases. This clearly demonstrates the rapid progress in this field. An example of the immediate implications of these contributions is that, until recently, the MS-based discrimination of the hairless skin and bone remains of sheep and goats was solely based on a single, relatively long, collagen peptide [43], [62] (Fig. 2), now this discrimination can be achieved on the basis of a much longer list of species diagnostic peptides as reported for sample 1 and 12 (Table S3, S4 and S5 in File S1). This improvement is partially due to the experimental setup adopted, however, most of the previously unreported diagnostic peptides were detected adopting a shotgun proteomics approach instead of focusing on the few most abundant proteins in bone, skin and hair. The availability of more markers enables a more secure identification of goatskin samples, which are closely related to sheep and equally present in the same regions and time periods as the samples analysed.

Apart from improving species identification, the availability of a reference proteome, or an extended protein list, for the most common domestic animals further enables the identification of proteins solely expressed in a specific tissue, developmental phase, or biological process [54]. For example, the detection of foetal haemoglobin, which is only expressed in animals younger than a few months [64], suggests that the cape from Møgelmose, was produced from a calf slaughtered within a few months of birth. A skin element from this cape was previously identified as the genus *Martes* [11], thus the present analysis represents crucial new information, also on preferences for specific qualities for production, as calfskin is much softer than skins from older animals. At this slaughter age, skin and meat of a higher quality would have been obtained. This result adds new perspectives to the interpretation of prehistoric animal husbandry and is highly pertinent to broader studies of animal bone assemblages. This type of information can only be provided by protein analysis as, while the genome of an organism is almost identical in all its tissues and developmental phases, its proteome can be developmental phase-specific. Our results demonstrate that MS-based ancient peptide sequencing is a reliable method for species identification, and yields information unobtainable with other methods.

Despite the novel, reliable results providing secure species assignment, the identification of archaeological skin garments based on MS-based ancient peptide sequencing also comes with certain limitations. In particular, reference protein databases are still incomplete, as exhaustive protein lists are at the moment only available for a limited number of species. This shortcoming, however, will eventually become less of an issue in the near future, as the rapid progress of genome-sequencing projects will soon make reference proteomes available for an increasing number of species, enabling even more secure species identification and higher taxonomical resolution. MS-analysis remains a (minimally) destructive approach, requiring sophisticated equipment and laboratory facilities. Consequently, it cannot be immediately available for all archaeological skin samples, and its diagnostic value is limited to the analysed skin element, which is only one among the many elements used to assemble a skin garment.

Although PMF-based approaches allow relatively rapid and inexpensive characterisation, thus making this approach ideal for large-scale applications and commercial quality control analyses, the maximisation of molecular recovery and data interpretation is crucial when applying even minimally destructive analyses to irreplaceable material of high cultural heritage value. Despite the necessity to sacrifice small parts of archaeological objects in the process, the collection and public sharing of the richest possible set of molecular information compatible with the technology and knowledge available at the time of analysis is of infinite value for the understanding of our distant past.

## Conclusions

The aim of this study was to compare established, morphology-based methods for species identification of archaeological skin objects from bogs with MS-based ancient peptide sequencing. The three methods adopted, in some cases, gave inconsistent results. Microscopy was challenged by general problems caused by degraded and partial fibres of archaeological material, while MS yielded secure peptide signals indicating that this method is suitable for application in the archaeological context examined. It thus represents a promising approach for future archaeological skin garment species identification. Although public databases of protein sequences are not yet complete, they already enable the determination of the most common domesticated species. Microscopy, on the other hand, holds the advantage of being relatively inexpensive, non-destructive, and easily applicable to a large number of samples, and sometimes, the sole option when dealing with mineralised samples. MS-based peptide sequencing could also be used to improve microscopy-based identification through the creation of reference collections of archaeological skin samples securely identified by peptide sequencing validation.

Based on the results presented here, it may be concluded that morphology-based species identification methods represent valid preliminary screening tools; however, for de-haired samples, or samples assigned by microscopy to species other than sheep, mass spectrometry-based peptide sequencing is highly recommended for achieving secure species identification.

## Supporting Information

File S1 **Supporting Information file containing supporting text, figures and tables.**
(PDF)
