# Supplementary material for: Species Identification of Archaeological Skin Objects from Danish Bogs: Comparison between Mass Spectrometry-Based Peptide Sequencing and Microscopy-Based Methods
Source: PLoS One. 2014 Sep 26;9(9):e106875. doi: 10.1371/journal.pone.0106875 (PMC4178020; doi:10.1371/journal.pone.0106875)
Supplement: File S1 — Supporting Information file containing supporting text, figures and tables. (PDF) [file pone.0106875.s001.pdf]

Supporting information for:

Species identification of archaeological skin objects from Danish bogs: comparison between mass spectrometry-based peptide sequencing and microscopy-based methods

Luise Ørsted Brandt[1,2], Anne Lisbeth Schmidt[3], Ulla Mannering[1,4], Mathilde Sarret[5], Christian D. Kelstrup[6], Jesper V. Olsen[6], Enrico Cappellini[2]

1 Centre for Textile Research, University of Copenhagen, Copenhagen, Denmark

2 Centre for GeoGenetics, University of Copenhagen, Copenhagen, Denmark

3 Department of Environmental Archaeology and Materials Science, The National Museum of Denmark, Copenhagen, Denmark

4 Department of Ancient Cultures in Denmark and the Mediterranean, The National Museum of Denmark, Copenhagen, Denmark

5 European School of Chemistry, Polymers and Materials Science, University of Strasbourg, Strasbourg, France

6 Novo Nordisk Foundation Center for Protein Research, University of Copenhagen, Copenhagen, Denmark

Table of contents

Text S1. Ancient peptide sequencing and identification: methods **3**
Mass Spectrometry-based peptide sequencing sample preparation, procedure A 3
LC-ESI high-resolution MS/MS analysis, procedure A 4
Mass Spectrometry-based peptide sequencing sample preparation, procedure B 4
NanoLC-ESI high-resolution MS/MS analysis, procedure B 6
Protein identification of spectra from procedures A and B 7

Supplementary figures **9**
Figure S1. The archaeological skin objects investigated. 9
Figure S2. SEM pictures of modern cross-sectioned hairs of the most common domesticated species used as reference material. 10
Figure S3. Example of evidence used for species identification by transmitted light microscopy. 11
Figure S4. Example of evidence used for species identification by SEM. 12
Figure S5. Examples of tandem spectra supporting the identification of sample n. 1 (Baunsø, NM D11103a) as goatskin. 13

Supplementary Tables **14**
Table S1. Morphological traits used for species identification of the commonest domesticated species by Method 2 (light microscopy and SEM). 14
Table S2. General statistics on protein and peptide recoveries for each sample. 15
Table S3. Peptides supporting the identification of Hemoglobin fetal subunit beta in sample 10 (Møgelmose, NM 16316). 15
Table S4. Proteins bearing observed species-diagnostic peptides. 18
Table S5. Species-diagnostic peptides observed. 23
Table S6. Statistics supporting the identification of the sheep/goat diagnostic collagen type-1 alpha-2 peptide. 24

Supplementary References **25**

Text S1. Ancient peptide sequencing and identification: methods

Mass Spectrometry-based peptide sequencing sample preparation, procedure A

For each ancient skin sample, two subsamples of 11-48mg were cut into small pieces with a scalpel. Each subsample was then transferred in a protein LoBind 1.5mL tube (Eppendorf, Germany). Each of the two subsamples was processed independently as follows. Each sample was re-suspended in 500µL of 100mM ammonium bicarbonate solution at pH 8.00, it was shaken vigorously for 1min and it was pelleted by centrifugation at 14000g for 5mins. The supernatant was discarded. This step was performed twice. The pellet was re-suspended in 1mL 1.2M HCl and incubated at 4˚C for 24h. It was then pelleted by centrifugation at 14000g for 10mins. The supernatant was collected and dried in a centrifugal evaporator for 20mins at room temperature. The pellet generated after evaporation was re-suspended in 200µL 50 mM ammonium bicarbonate pH 8.00. The pH was checked using pH-indicator strips and adjusted to 8.00 with concentrated ammonium hydroxide and the solution was incubated at 70˚C for 24h. After centrifugation at 14000g for 10mins, to precipitate any eventual insoluble residue, the supernatant was collected and transferred in a fresh protein LoBind 1.5mL tube. Digestion was then started adding 4µL of 0.5µg/µL sequencing grade trypsin solution (Promega, Nacka Sweden) and incubating at 37˚C overnight. The following morning 2µL of fresh trypsin were added and digestion was extended for 6 additional hours. Digestion was quenched with 10% trifluoroacetic acid to a final concentration of 0.2-0.8% to reach pH < 2.00 and tryptic peptides were immobilised on C18 stage tips, as described in procedure B.

LC-ESI high-resolution MS/MS analysis, procedure A

All peptide mixtures were analysed by online nanoflow reversed-phase C18 liquid chromatography tandem mass spectrometry (LC-MS/MS) as described previously [1].

Mass Spectrometry-based peptide sequencing sample preparation, procedure B

Generation of tryptic peptides from the second group of archaeological bog skin samples was performed using a filter-aided sample preparation (FASP) protocol [2], modified for ancient and degraded samples [3]. For each ancient skin sample a subsample of 8-53mg was cut into small pieces with a scalpel and transferred in a protein LoBind 1.5mL tube (Eppendorf, Germany). The sample was suspended in a 300μL lysis buffer (4% SDS, 0.1M DTT, 0.1M Tris/HCl, pH 8.00), manually homogenised using a sterile micropestle (Eppendorf, Germany) and thereafter heated to 95°C under agitation for 10mins. The samples were then centrifuged at 14000g for 10mins to precipitate insoluble debris. The supernatant (SDS-fraction) was mixed with 2mL 8M urea in 0.1 M Tris/HCl pH 8.00 and ultrafiltered at 4500g, for the time necessary to remove all the solvent except what remained in the dead volume, through an Amicon Ultra-4 (Merck Millipore, USA) centrifugal filter unit with 10kDa NMWL. Sample preparation continued with washing the fraction retained above ultrafilter with 2mL 8M urea in 0.1M Tris/HCl pH 8.00. Alkylation was achieved by re-suspending the fraction retained above ultrafilter in 500μL of 50mM 2-Chloroacetamide (CAA), 8M urea 0.1M Tris/HCl pH 8,00 and incubating for 20mins in the dark at room temperature. CAA was removed by washing with 1mL of 8M urea 0.1M Tris/HCl pH 8.00 twice, finally urea was removed by washing twice with 1mL 50mM ammonium bicarbonate, pH 7.50-8.00. The fraction retained above ultrafilter was re-suspended in 300μL of 50mM ammonium bicarbonate pH 7.50-8.00 and mixed. One

microliter of solution was collected for protein quantification using a Qubit fluorometer (Invitrogen-Life Technologies, USA).

Protein digestion was started by adding 4µL of 0.5µg/µL sequencing grade trypsin solution. After mixing, pH was checked using pH strips and, when necessary, adjusted to 7.50-8.00. The ultrafiltration units were transferred into new 15mL tubes, sealed with parafilm and incubated overnight at 37˚C. The following morning two supplemental microliters of fresh trypsin solution 0.5µg/µL were added and digestion was extended for an additional 6 hours. Ultrafiltration units were then centrifuged at 4500g for 10mins to collect the digested peptides and 1µL of the filtrate was collected for Qubit protein quantification. An additional 500µL aliquot of 50mM ammonium bicarbonate pH 7.50-8.00 was added to the filter, mixed, and centrifuged at 4500g for 10mins to elute possible remaining peptides. The filtrate was then transferred to a 1.5mL protein LoBind tube and acidified with 10% trifluoroacetic acid to a final concentration of 0.2-0.8% to reach pH < 2.00. C-18 solid phase extraction (SPE) Stage tips were prepared in-house and sequentially conditioned with 150µL methanol, then 150µL 80% acetonitrile solution (80% acetonitrile, 0.5% acetic acid, 19.5% ddH_2O -v/v/v-), and finally 150µL 0.5% acetic acid in ddH_2O (v/v) [1,4] The acidified peptides were then loaded into the stage-tips and immobilized onto the C-18 filter by centrifugation. Next, the filter was washed with 150µL 0.5% acetic acid in ddH_2O, centrifuged until dry, and stored at -20˚C. Immediately before LC-MS/MS analysis, tryptic peptides were eluted from the Stage-tips membrane, using 10µL of three solutions of progressively more concentrated acetonitrile (40-60-80%, 0.5% acetic acid, in ddH_2O -v/v/v-), directly into a 96-well plate. The samples in the plate were concentrated in a centrifugal evaporator to a volume of approx. 2-4µL and re-suspended in 1% TFA to reach a final volume of 10µL.

NanoLC-ESI high-resolution MS/MS analysis, procedure B

The LC-MS system consisted of an EASY-nLC system (Thermo Scientific, Odense, Denmark) interfaced to the Q Exactive (Thermo Scientific, Bremen, Germany) through a nano electrospray ion source. For each peptide sample 5µL were auto-sampled onto and directly separated in a 15cm analytical column (75µm inner diameter) in-house packed with 3µm C18 beads (Reprosil-AQ Pur, Dr. Maisch) with a 65mins linear gradient from 5% to 26% acetonitrile followed by a steeper linear 14min gradient from 26% to 48% acetonitrile. Throughout the gradients a fixed concentration of 0.5% acetic acid and a flow rate of 250nL/min were set. A final washout and column re-equilibration added an additional 11mins to each acquisition. The effluent from the HPLC was directly electrosprayed into the mass spectrometer by applying 2.0kV through a platinum-based liquid-junction. The Q Exactive was operated in data-dependent mode to automatically switch between full scan MS and MS/MS acquisition. Software control was Tune version 2.0-1428 and Excalibur version 2.2.42. The settings used were as described for the "sensitive" acquisition described by Kelstrup et *al*. [5]. Each full scan MS was followed by up to 10 MS/MS events. The isolation window was set at 2Th and a dynamic exclusion of 90 seconds was used to avoid repeated sequencing. Only precursor charge states above 1 and below 7 were considered for fragmentation. A minimum intensity threshold for triggering fragment MS/MS was set at 1e5. Full scan MS were recorded at a resolution of 70,000 at m/z 200 in a mass range of 300-1700 m/z with a target value of 1e6 and a maximum injection time of 20 ms. Fragment MS/MS were recorded with a fixed ion injection time set to 120 ms through a target value set to 1e6 and recorded at a resolution of 35,000 with a fixed first mass set to 100 m/z.

Protein identification of spectra from procedures A and B

Raw files generated during spectra acquisition were searched on a workstation using the MaxQuant algorithm v. 1.2.2.5 [6] and the Andromeda peptide search engine [7] initially against a target/reverse custom-made list of all the mammalian Alpha-1 type I, Alpha-2 type I and Alpha-1 type III collagen sequences publicly available in UniProt and nrNCBI (approximately 240 accessions in total), and then against the target/reverse protein list in the *Bos taurus* and *Homo sapiens* reference proteomes (34365 and 84946 -including isoforms- accessions downloaded from UniProtKB on Oct. 24 2012 and Dec. 31 2012, for *B. taurus* and *H. sapiens* respectively) together with the complete list of proteins available in NCBI RefSeq after taxonomical restriction to *Ovis aries* (22752 accessions, downloaded on Jan. 8, 2013) and *Capra hircus* (31461 accessions, downloaded on Dec. 2, 2013). In every search, spectra were also matched against the common contaminants, such as wool keratin and porcine trypsin sequences, downloaded from Uniprot.

Trypsin was selected as the proteolytic enzyme and two missed cleavages were allowed. Oxidation (M and P), deamidation (N and Q), acetylation (K), G→pyro-Glu (N-term Q), and E→pyro-Glu (N-term E) were selected as variable amino-acid modifications. Carbamidomethylation (C) was selected as fixed modification. Default values were used for precursor (6 ppm) and fragment (20 ppm) ions mass tolerance. False-discovery rate was set at 1% and minimum peptide-score and peptide sequence length were set at 80 and 6 respectively. The amount of random matches was evaluated performing MaxQuant search against reversed sequences. For the identification of species-diagnostic peptides, possible environmental contaminants, such as actin, tubulin, keratins and keratin-associated proteins, as well as proteins commonly used in mass spectrometry facilities as standards or calibrants and proteins highly conserved, such as histones, and all human

proteins were excluded from further investigation. A minimum of two unique peptides was required for confidently calling proteins. Using locally installed, command line operated, Blastp algorithm, part of the stand-alone BLAST suite "BLAST+" [8], protein-unique peptides from this subset of identified proteins were remotely aligned, -remote, against the entire nrNCBI protein database, -db nr, using optimization for short sequences, -task blastp-short, allowing no gaps, -ungapped, and not applying composition-based statistics, -comp_based_stats F. Those peptides presenting 100% alignment, or alternatively a fragmentation pattern exclusively compatible with either *Ovis, Capra* or *Bos* genus among mammals (after excluding any possible I/L isobaric substitution, but contemplating possible deamidation events), and excluding any possible origin from biological contaminants in soil (i.e. bacteria and fungi), were reported as species-diagnostic for either *Ovis aries, Capra hircus* or *Bos taurus*. We also identified "semi-specific" peptides not exclusively matching sheep/goat or cattle, when the nature of the sample excluded any other plausible alternative assignment.

Supplementary figures

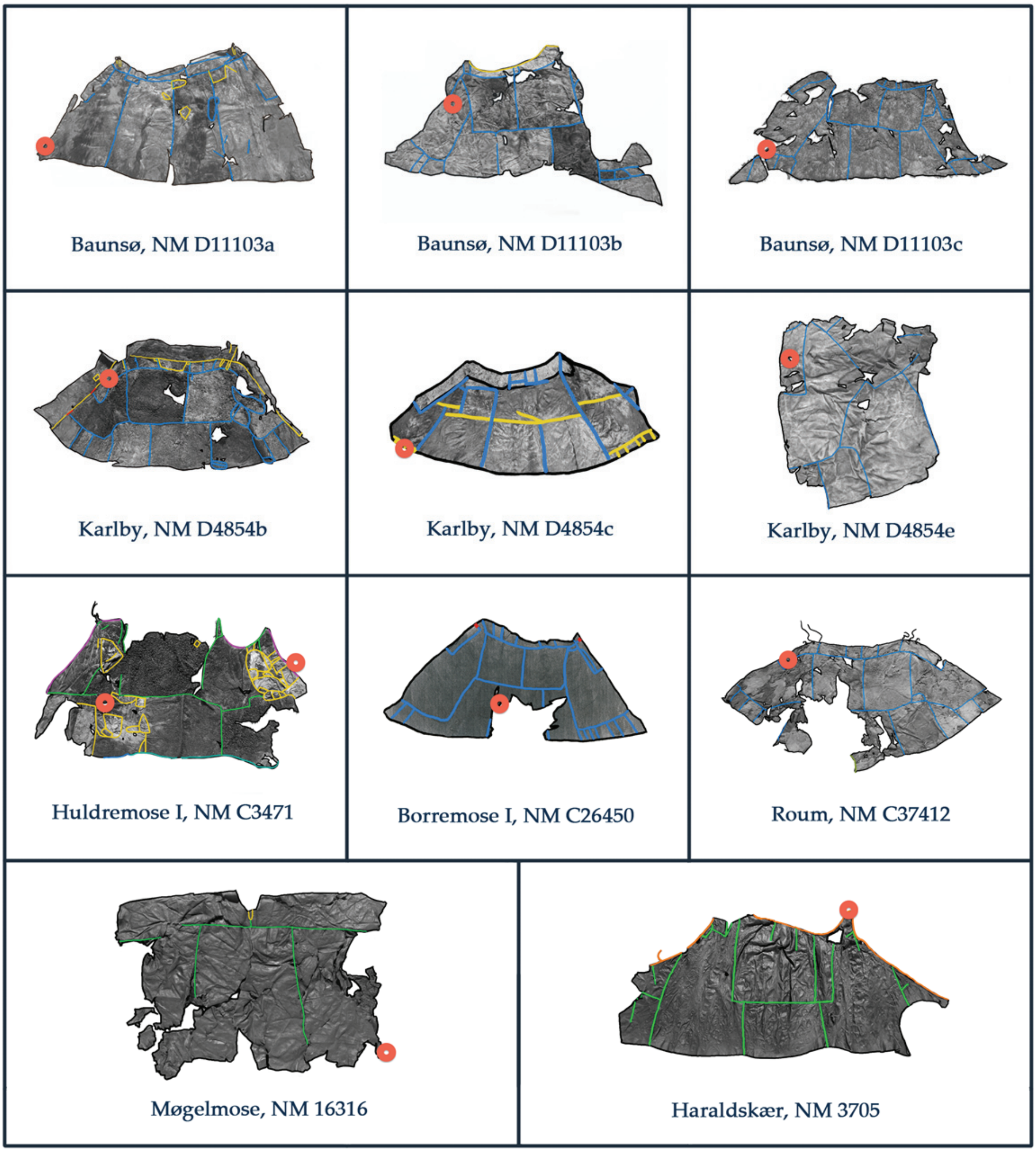

Figure S1. The archaeological skin objects investigated. Coloured lines indicate the seams joining skin elements composing each garment. Red rings indicate the sampling points for all three analyses. For the Huldremose I cape, two elements were sampled. The sample referred to as “dark” is the one on the right. Photos by Roberto Fortuna, the National Museum of Denmark.

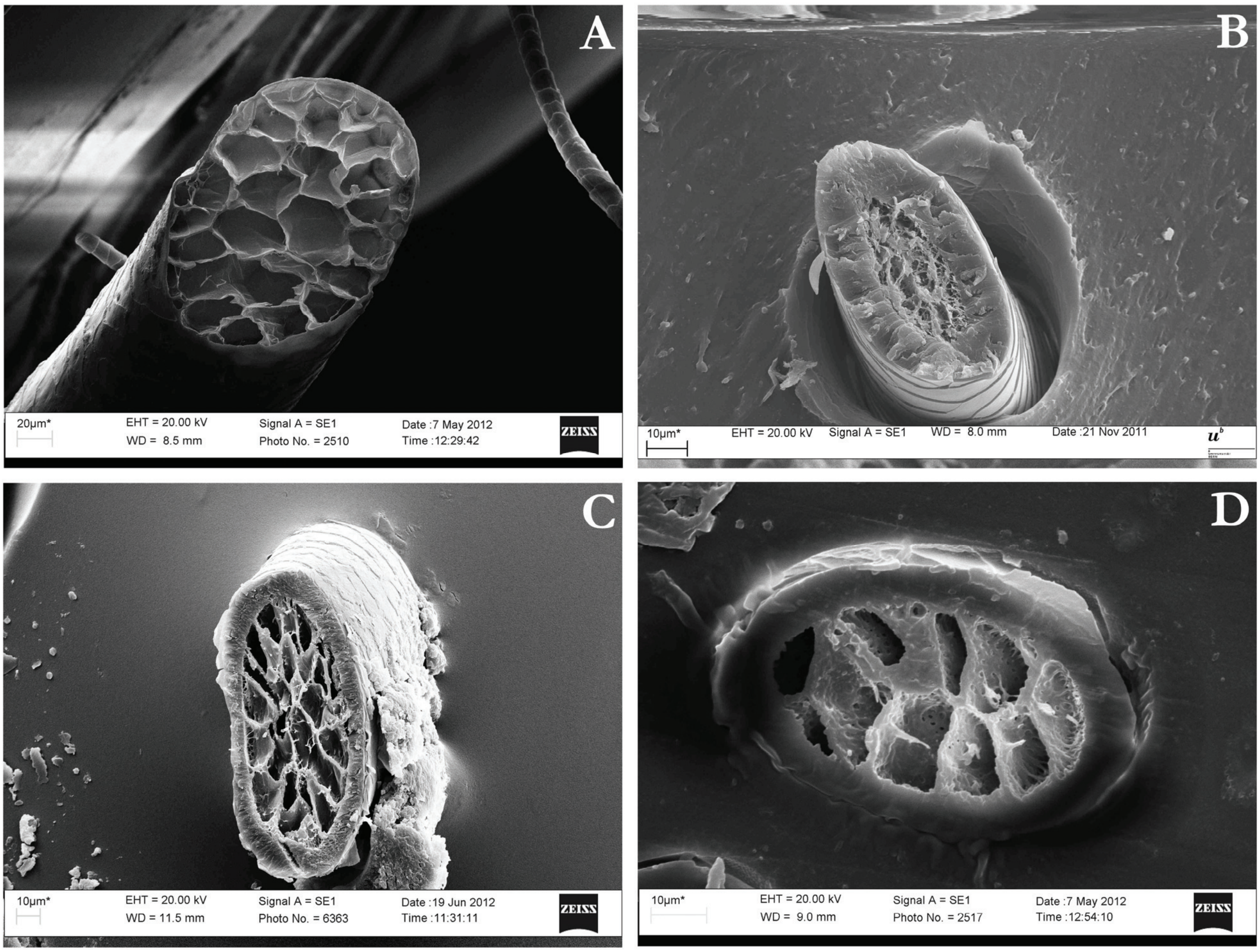

Figure S2. SEM pictures of modern cross-sectioned hairs of the most common domesticated species used as reference material. (A) sheep, (B) cattle, (C) goat, (D) horse.

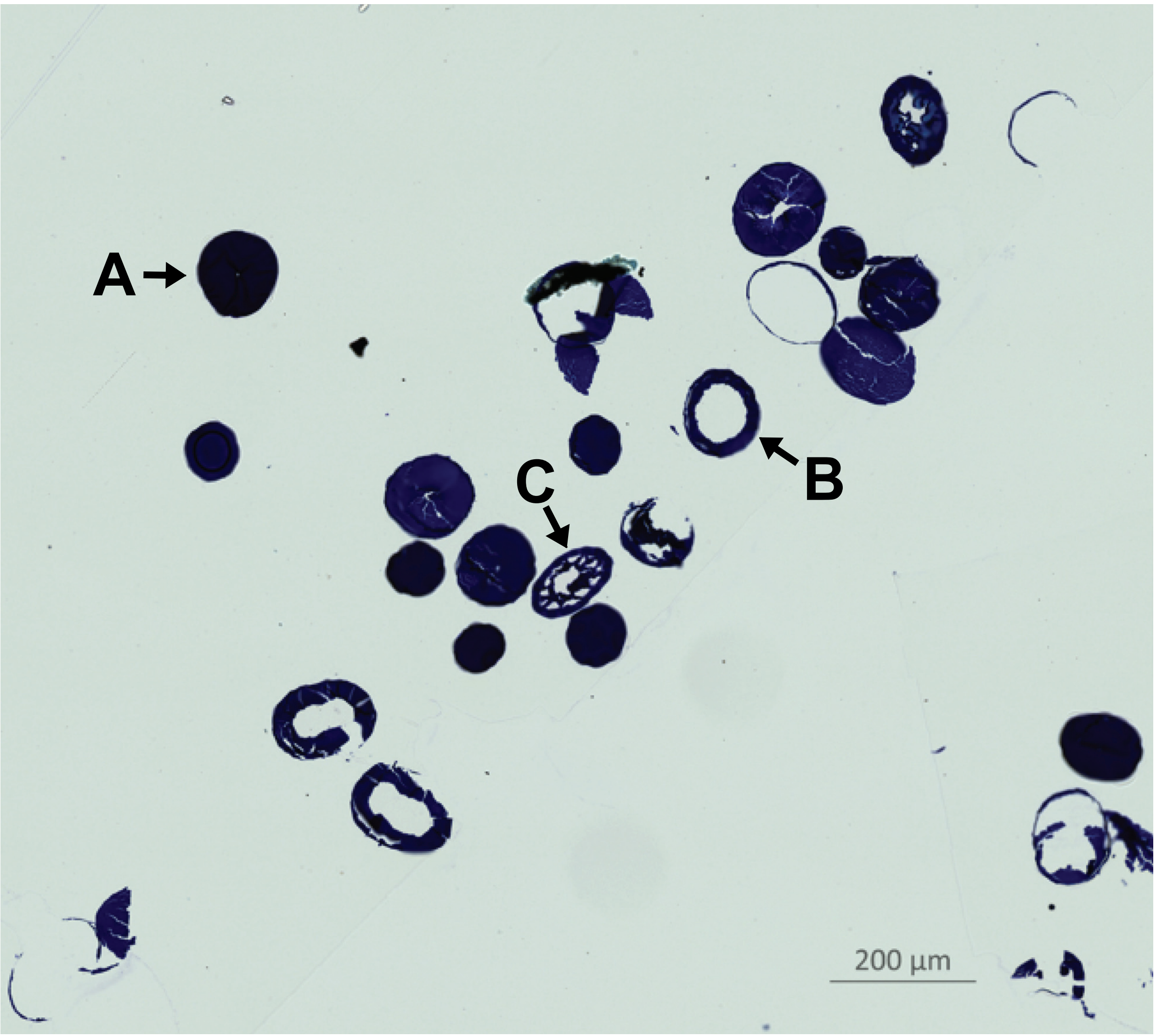

Figure S3. Example of evidence used for species identification by transmitted light microscopy. Microscopy of the cross-section of hairs from sample 1 shows three different variants of guard hair: (A) round hair with continuous medulla, approximately 80-130 µm wide. The medulla displays wavy lines and an unfilled center without pigmentation. The cuticular outer layer poorly preserved; (B) round to oval hair with absent medulla, approximately 100 µm wide; cortex approximately 30 µm thick. The cuticular outer layer poorly preserved; (C) round to oval hair with open, wide lattice medulla, approximately 100 µm wide. The cuticular outer layer poorly preserved. The size and shape of hair as well as the absence of medulla in some hairs indicated cattle, when compared to modern cattle hair [9-16] and present-day contemporary samples.

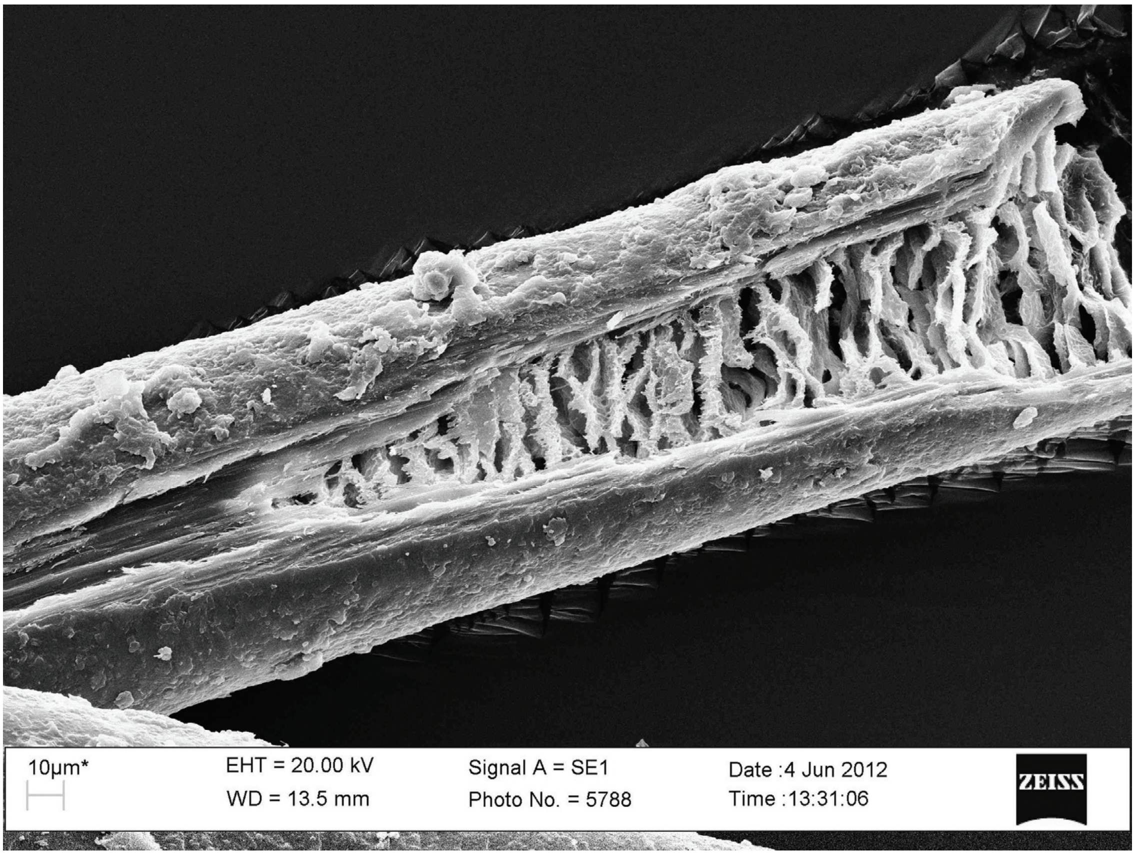

Figure S4. SEM of hair for species identification by Method 2. For sample 1, scales are poorly preserved or missing. When the medulla is preserved, the cross-section shows fields with a dot-like structure. Compared to the sample displayed, modern sheep have a much finer epidermis and do not display the dot-like structure of medulla chambers. Cattle have very fine "bubbles" filling in the medulla, which appear very different from the sample. Goats and horses have a similar epidermis size and medulla, including a dot-like structure in the medulla chambers. Goats and horses can be difficult to distinguish, especially if one or more of the three criteria looked for are poorly preserved. The sample was designated as horse skin by light microscopy, as the fine lines at the edge of the medulla before the epidermis, specific for goats, were not visible.

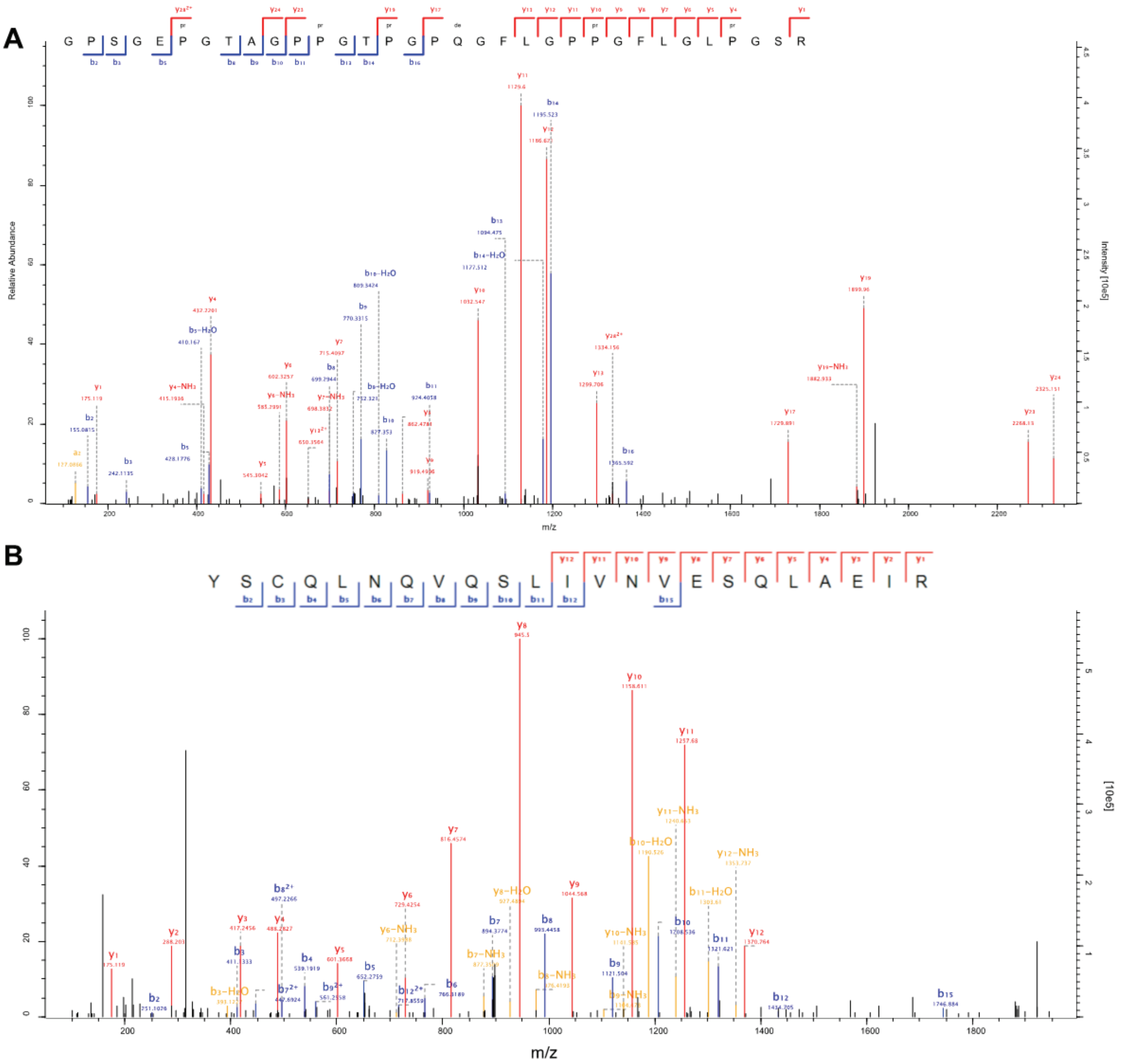

Figure S5. Examples of tandem spectra supporting the identification of sample 1 as goatskin. (A) Type I alpha-2 collagen (COL1A2) diagnostic peptide in the goat-diagnostic version [17,18] and (B) peptide belonging to keratin33A previously assigned to goat [19].

Supplementary Tables

Fibre	Scales of primary hairs	Cross section	Medulla
SHEEP fine 10-20um, large up to 150um	mosaic, straight margins	round-oval<br>fine cortex	cloisonné
GOAT fine 10-15um, large up to 200um	rippled, fine horizontal, intervals, 5-10um	oval or kidney-shape<br>SEM: cloisonné	SEM: cloisonné<br>LM: flatten disk<br>margins fringed<br>often large and dark
HORSE fine 10-20um, large up to 120um	fine straight or rippled, intervals 10um	oval<br>LM: medulla „star“<br>SEM: cloisonné	LM: disk-fields<br>SEM: cloisonné<br>margins flat<br>mane: irregular, small
CATTLE fine: 20 um, large	straight-wave, wide intervals, 10-15um	round-oval<br>SEM: cloisonné	granulated (LM/SEM)<br>irregular width

Table S1. Morphological traits used for species identification of the commonest domesticates by Method 2 (light microscopy and SEM). The “X” symbol, in red, indicates cases in which a morphological trait differs sufficiently to distinguish between two species. The “Z” symbol, in blue, indicates cases where two species share a morphological trait. The table illustrates how species are identified based on morphological characteristics of the medulla, cross-section and scales, and demonstrates where the species differ and where similarities cause problems in species identification. For example, sheep and goat can only be differentiated by the appearance of the primary follicles as the fine fibres are too similar. Evaluation of scale height, which is used in today’s industry to differentiate sheep wool from cashmere, is only possible in extremely well preserved archaeological samples. Distinguishing the medulla of sheep and goat hairs is difficult as it is often either degraded or the fibres are too darkly stained to make them visible under light microscopy. On the other hand, the table shows that cattle can be distinguished from the other species by their granulated medulla, which is visible on cross-sectioned fibres using light microscopy or in SEM if the fibres are not too dark.

Sample no.	Sample	Recovery (mg pt/ g sample)	Total ident. prot.	Total ident. pept.*	Total ident. MS/MS spectra*	Prot. after filtering	Unique pept. after prot. filtering	MS/MS spectra after prot. filtering**
1	Baunsø NM D11103a		110	655 (367| 521)	31730 (16513+15217)	18	55	213
2	Baunsø NM D11103b	1.32	29	144	32029	2	33	56
3	Baunsø NM D11103c	2.59	42	201	30497	5	26	41
4	Borremose I NM C26450		108	739 (400|605)	37770 (20491+17279)	18	81	437
5	Huldremose I NM C3471 dark		119	841 (477|662)	42828 (21246+21582)	23	140	932
6	Huldremose I NM C3471 light		132	772 (379|603)	44570 (21940+22630)	26	65	240
7	Karlby NM D4854b	20.13	107	528	33796	18	81	225
8	Karlby NM D4854c	7.54	77	368	34781	14	46	110
9	Karlby NM D4854e	10.00	55	243	27168	5	25	53
10	Møgelmose NM 16316		139	739 (373|559)	38656 (16179+22477)	23	66	194
11	Roum NM C37412	8.52	13	81	21541	1	12	17
12	Haraldskær C3705		108	678 (321|583)	38108 (19161+18947)	23	78	369
	Modern sheep		362	1078	11728	127	437	3679
	Modern goat		273	779	9664	66	273	3206
	Modern cattle		177	480	2366	28	123	462

Table S2. General statistics on protein and peptide recoveries for each sample. *Including contaminants. ** Relative to unique peptides. Protein recoveries were measured only for skin samples prepared with procedure B.

Accession number	Protein Name	Unique Peptides	Uniq. Pep. Seq. Cov. [%]	Seq. Length	Matched spectra	
P02081	Hemoglobin fetal subunit beta	3	17.9	201	18	
	Sequence	Length	Mass	Charges	MaxQuant Score	Matched spectra
	AAVTSLFAK	9	906,51747	2	91.318	1
	FGSEFSPELQASFQK	15	1700,8046	2	102.73	1

Table S3. Peptides supporting the identification of Hemoglobin fetal subunit beta in sample 10 (Møgelmose, NM 16316).

Sample	No.	Accession number	Protein Name	All Matching Peptides*	Unique Peptides	Total Seq. Cov. [%]**	Uniq. Pep. Seq. Cov. [%]	Seq. Length	Matched spectra **
Baunsø NM D11103a	1	NP_001272646.1	hair acidic keratin 1	34	1	64.4	5.6	413	394
	2	XP_005680154.1	keratin. type II cuticular Hb1-like	42	5	73.6	10.4	402	382
	3	XP_005693870.1	keratin. type I cuticular Ha4-like	36	4	60.7	8.8	433	456
	4	BAJ65377.1	keratin33A	29	1	59.7	5.7	404	395
Borremose I NM C26450	1	gi|426220723	collagen alpha-1(III)	31	7	29	9.4	1467	170
Haraldskær NM C3705	1	gi|426227338	collagen alpha-2(I)	56	23	50.1	24.6	1364	458
	2	XP_005693869.1	keratin. type I cuticular Ha8-like	12	5	28.8	13.9	476	47
	3	NP_001272646.1	hair acidic keratin 1	35	2	67.1	5.6	413	791
	4	XP_005678993.1	collagen alpha-2(I) chain	60	60	57.3	57.3	1364	445
	5	NP_001272697.1	keratin associated protein 12.1	2	2	19	19	116	2
	6	XP_005680154.1	keratin. type II cuticular Hb1-like	41	5	72.1	10.4	402	801
	7	BAJ65365.1	keratin33A	34	1	72	4.2	404	738
	8	XP_005693870.1	keratin. high-sulphur matrix protein. IIIA3-like	36	4	65.8	10.4	433	874
Huldremose I NM C3471 dark skin	1	gi|426227338	collagen alpha-2(I)	41	29	41.5	31.9	1364	301
	2	gi|426220723	collagen alpha-1(III)	30	5	30.9	6.7	1467	169
Huldremose I NM C3471 light skin	1	gi|426220723	collagen alpha-1(III	43	8	40.8	10.5	1467	218
Møgelsmose NM 16316	1	P02465	collagen alpha-2(I)	51	6	51.2	7	1364	361
	2	Q2KJ32	Selenium-binding protein 1	4	3	10.2	7.8	472	9
Karlby NM D4854b	1	gi|426220723	collagen alpha-1(III)	49	13	47.6	15.1	1467	125
	2	gi|426237753	collagen alpha-1(I)	69	2	58.9	4	1471	338
Karlby NM D4854c	1	gi|426220723	collagen alpha-1(III)	36	9	38.4	11.5	1467	109

Karlby NM D4854e***	1	gi|426227338	collagen alpha-2(I)	28	16	33.1	23.2	1364	55
Roum NM C37412***	1	gi|426227338	collagen alpha-2(I)	15	12	16.9	13.9	1364	21
Baunsø NM D11103b	1	P02465	collagen alpha-2(I)	31	8	34	8.8	1364	64
Baunsø NM D11103c	1	gi|426227338	collagen alpha-2(I)	41	14	41.1	18	1364	77
	2	gi|426237753	collagen alpha-1(I)	55	3	50.1	5.4	1471	142
Modern sheep	1	gi|426230702	perilipin-4	4	4	5.1	5.1	1051	4
	2	gi|426219699	major allergen Equ c 1-like	4	4	18.1	18.1	182	8
	3	gi|426220721	collagen alpha-2(V) chain	24	23	30.5	29.8	1499	47
	4	gi|426259137	serpin A3-1-like. partial	3	2	13.2	9.8	204	4
	5	gi|426241977	uncharacterized protein LOC101116248	2	2	6.7	6.7	564	6
	6	gi|426251176	tenascin-X	13	4	6.4	1.6	3949	16
	7	gi|426258629	complement C3-like	8	2	14.9	4.1	657	13
Modern goats	1	AAX45026.1	immunoglobulin gamma-1 chain F7-299	1	1	3.8	3.8	239	1
	2	XP_005701292.1	odorant-binding protein-like	2	2	17.4	17.4	172	2
	3	XP_005678993.1	collagen alpha-2(I) chain	76	76	70.8	70.8	1364	285
	4	XP_005679869.1	lumican	14	5	37.4	12.6	342	58
	5	XP_005695453.1	alpha-1-antiproteinase-like	7	2	22.3	7.3	381	14
	6	XP_005701928.1	serpin A3-1-like	6	1	29.3	9.1	164	11
	7	ACH86010.1	II alpha globin	3	1	21.8	10.6	142	3
	8	XP_005692313.1	major allergen I polypeptide chain 1-like	1	1	16.1	16.1	93	2
Modern cattle	1	F1MYG5	prelamin-A/C	6	6	11.3	11.3	664	7
	2	F1MJB5	filaggrin	2	2	12.6	12.6	406	2
	3	P02465	collagen alpha-2(I) chain	45	8	48.1	9.1	1364	96

4	Q28133	allergen Bos d 2	4	3	18	13.4	172	4
5	E1BB91	collagen. type VI. alpha 3	24	24	10.6	10.6	3170	34

Table S4. Proteins bearing observed species-diagnostic peptides. * Including non-unique peptides. ** Based on all matching peptides. *** For samples 9 (Karlby NM D4854e) and 11 (Roum NM C37412) no single peptide allowed to discriminate sheep from goats. Proteins bearing peptides supporting ovine identification are reported.

Sample	Accession number	Protein name	N.	Sequence	Length (aa)	Mass	Charge	MaxQuant Score	Matched spectra	References.
No 1. Baunsø NM D11103a	NP_001272646.1	hair acidic keratin 1	1	GLLDSEDCKLPCNPCATTNAYGK	23	2583.1404	3	82.475	1	
	XP_005680154.1	keratin. type I cuticular Ha4-like	1	KKYEEEIALR	10	1277.698	3	129.39	1	
			2	LEAAVTQAEQQGEAALNDAKR	21	2212.1084	3	156.67	3	
	XP_005693870.1	keratin. type I cuticular Ha4-like	1	SDLEAQVESLKEELLFLKK	19	2218.2097	3.4	104.84	4	
	BAJ65377.1	keratin33A	1	YSCQLNQVQSLIVNVESQLAEIR	23	2690.3698	3	150.36	6	[19]
No. 2. Baunsø. NM D11103b	P02465	collagen alpha-2(I)	1	GAPGAIGAPGPAGANGDR	18	1504.7383	2.3	132.05	3	
			2	GYPGNAGPVGAAGAPGPQGPVGPVGK	26	2228.1338	2	119.83	1	
			3	HGNRGEPGPAGAVGPAGAVGPR	22	1980.0038	3.4	176.46	3	
			4	IGQPGAVGPAGIR	13	1191.6724	2	113.77	2	Peptide A [17,18]
			5	SGETGASGPPGFVGEK	16	1475.6892	3	111.74	1	
No. 3. Baunsø. NM D11103c	gi|426237753	collagen alpha-1(I)	1	AGEVGPPGPPGPAGEKGAPGADGPAGAPGTPGPQGIAGQR	40	3453.7025	4	167.95	1	
No. 4. Borremose I. NM C26450	gi|426220723	collagen alpha-1(III)	1	GFPGNPGPPGSPGPAGHQGAVGSPGPAGPR	30	2630.2738	3	91.175	1	
No. 5. Huldremose I dark. NM C3471	gi|426227338	collagen alpha-2(I)	1	TGQPGAVGPAGIR	13	1179.636	2.3	110.64	11	Peptide A [17,18]
	gi|426220723	collagen alpha-1(III)	1	GFPGNPGPPGSPGPAGHQGAVGSPGPAGPR	30	2630.2738	3	137.16	1	

No. 6. Huldremose I light. NM C3471	gi|426220723	collagen alpha-1(III)	1	GFPGNPGPPGSPGPAGHQGAVGSPGPAGPR	30	2630.2738	3.4	124	9	
No. 7. Karlby. NM D4854b	gi|426220723	collagen alpha-1(III)	1	GFPGNPGPPGSPGPAGHQGAVGSPGPAGPR*	30	2630.2738	2.3	183.25	7	
	gi|426237753	collagen alpha-1(I)	1	AGEVGPPGPPGPAGEKGAPGADGPAGAPGTPGPQGIAGQR	40	3453.7025	3	211.59	1	
No. 8. Karlby. NM D4854c	gi|426220723	collagen alpha-1(III)	1	GFPGNPGPPGSPGPAGHQGAVGSPGPAGPR*	30	2630.2738	3	125	1	
No. 9. Karlby. NM D4854e**	gi|426227338	collagen alpha-2(I)	1	GEPGPVGAVGPAGAVGPR	18	1543.8107	2.3.4	161.8	5	
			2	GYPGNAGPVGAAGAPGPQGPVGPTGK	26	2230.1131	2.3	121.33	2	
			3	TGEPGAAGPPGFVGEK	16	1469.7151	3	106.42	1	
			4	TGPPGPAGISGPPGPPGPAGKEGLR	25	2220.1651	2	116.48	1	
			5	TGQPGAVGPAGIR	13	1179.636	2.3	118.88	3	Peptide A [17,18]
No. 10. Møgelmose. NM 16316	P02465	collagen alpha-2(I)	1	GAPGAIGAPGPAGANGDR	18	1504.7383	2	125.22	3	
			2	GDIGSPGRDGAR	12	1156.5585	2	134.38	1	
			3	HGNRGEPGPAGAVGPAGAVGPR	22	1980.0038	2.3	200.45	2	
			4	IGQPGAVGPAGIR	13	1191.6724	2.3	106.49	2	Peptide A [17,18]
			5	SGETGASGPPGFVGEK	16	1475.6892	2	92.063	1	
	Q2KJ32	Selenium-binding protein 1	1	VQTLTLQDGLIPLEIR	16	1808.0407	2.3	123.29	6	
No. 11. Roum. NM C37412**	gi|426227338	collagen alpha-2(I)	1	GEPGPVGAVGPAGAVGPR	18	1543.8107	2.3	145.55	3	
			2	GYPGNAGPVGAAGAPGPQGPVGPTGK	26	2230.1131	2	87.511	1	
			3	TGEPGAAGPPGFVGEK	16	1469.7151	3	93.237	1	
			4	TGPPGPAGISGPPGPPGPAGK	21	1764.9159	2	118.66	1	

			5	TGQPGAVGPAGIR	13	1179.636	2	112.02	2	Peptide A [17,18]
No. 12 Haraldskær. NM 3705	gi|426227338	collagen alpha-2(I)	1	TGQPGAVGPAGIR	13	1179.636	2	124.6	8	Peptide A [17,18]
	XP_005693869.1	keratin. type I cuticular Ha8-like	1	FGIELAQMQTLISNVEEQLSEIR	23	2647.3527	3	115.54	3	
			2	LAVEEDLCGLHK	12	1382.6864	3	102.4	1	
	NP_001272646.1	hair acidic keratin	1	GLLDSEDCKLPCNPCATTNAYGK	23	2583.1404	3.4	115.54	2	
			2	LPCNPCATTNAYGK	14	1565.6966	2	90.71	1	
	XP_005678993.1	collagen alpha-2(I) chain	1	GPSGEPGTAGPPGTPGPQGFLGPPGFLGLPGS	33	3012.5094	3	123.31	9	
	NP_001272697.1	keratin associated protein 12.1	1	IVYVIPSCQSSR	12	1407.718	2	80.69	1	
			2	PVLYVPVCYK	10	1236.6577	2	82.287	1	
	XP_005680154.1	keratin. type II cuticular Hb1-like	1	KKYEEEIALR	10	1277.698	2.3	178.54	2	
			2	LEAAVTQAEQQGEAALNDAKR	21	2212.1084	3.4	159.14	3	
	BAJ65365.1	keratin33A	1	QNHEQEVNTLQSQLGDR	17	1994.9406	2.3	221.47	11	
	XP_005693870.1	keratin. high-sulphur matrix protein. IIIA3-like	1	SDLEAQVESLKEELLFLK	18	2090.1147	2.3	165.9	6	
Modern sheep	gi|426230702	perilipin-4	1	DVSSQPEEAAAGEVPATGALSR	22	2141.0237	2	90.464	1	
	gi|426219699	major allergen Equ c 1-like	1	ENIIDLTR	8	972.52401	2	106.36	1	
	gi|426220721	collagen alpha-2(V) chain	1	GETGPPGPIGSQGLPGAVGTDGTPGAK	27	2374.1765	2.3	137.32	2	

	gi|426259137	serpin A3-1-like. partial	1	IFADADLSGITGTR	14	1435.7307	2.3	167.14	2
	gi|426241977	uncharacterized protein LOC101116248	1	ISLPFVNSSVSSLTVPSSNIR	21	2203.1848	3	92.474	1
	gi|426251176	tenascin-X	1	LGPISTEGSTAPLEK	15	1498.7879	2	96.591	1
	gi|426258629	complement C3-like	1	LVAYYTLSNANGQR	14	1568.7947	2	83.617	1
			2	VPINDGNGEAILR	13	1366.7205	2	78.884	1
Modern goat	AAX45026.1	immunoglobulin gamma-1 chain F7-299	1	ALEWLGGIR	9	1013.5658	2	105.52	1
	XP_005701292.1	odorant-binding protein-like	1	GDENTLLTHTVNVDEHGK	18	1977.9392	3	151.41	1
	XP_005678993.1	collagen alpha-2(I) chain	1	GPSGEPGTAGPPGTPGPQGFLGPPGFLGLPGSR	33	3012.5094	2.3	264.36	7
	XP_005679869.1	lumican	1	SLEYLDLSFNQITK	14	1669.8563	2.3	239.98	7
	XP_005695453.1	alpha-1-antiproteinase-like	1	VFSNGADLSGITEEQPLK	18	1903.9527	2.3	178.43	8
	XP_005701928.1	serpin A3-1-like	1	VFTSEADLSGITGVR	15	1550.794	2.3	128.15	2
	ACH86010.1	II alpha globin	1	VGSNAGAYGTEALER	15	1493.711	2	107.84	1
	XP_005692313.1	major allergen I polypeptide chain 1-like	1	YNQNPDVLETADILK	15	1731.8679	2.3	120.92	2
Modern cattle	F1MYG5	prelamin-A/C	1	ASASSGAQVGGSISSGSSASSVTVTR	26	2297.1095	2.3	181.69	2

F1MJB5	filaggrin	1	ESSVSQASDSEGYSGDVGR	19	1915.8032	2	187.56	1	
P02465	collagen alpha-2(I) chain	1	GAPGAIGAPGPAGANGDR	18	1504.7383	2	96.993	1	
		2	GEPGPAGAVGPAGAVGPR	18	1515.7794	2.3	151.18	3	
		3	HGNRGEPGPAGAVGPAGAVGPR	22	1980.0038	4	175.32	2	
		4	IGQPGAVGPAGIR	13	1191.6724	2	160.88	2	Peptide A [17,18]
		5	SGETGASGPPGFVGEK	16	1475.6892	2.3	190.24	3	
Q28133	allergen Bos d 2	1	GTSFTPEELEK	11	1236.5874	2	82.261	1	
E1BB91	collagen. type VI. alpha 3	1	LQASVTPLTTPVVSSK	16	1626.9192	2	116.96	1	
		2	QASMDNVK	8	891.41202	2	138.78	2	

Table S5. Species-diagnostic peptides observed. *Peptides matching non-uniquely with the assigned species in the nrNCBI protein database, but incompatible with any other assignments due to the nature of the sample. ** For samples 9 (Karlby NM D4854e) and 11 (Roum NM C37412) no single peptide allowed to discriminate sheep from goats. Peptides supporting ovine identification are reported. References reporting previously identified peptides are cited.

Sample No.	Sample	Peptide version	Mass	Charges	MaxQuant Score	Matched spectra
1	Baunsø NM D11103a	Goat	3012,5094	3	139.67	3
4	Borremose I NM C26450	Sheep	2952,5094	3,4	148.72	14
5	Huldremose I NM C3471 dark	Sheep	2952,5094	3	142.28	6
6	Huldremose I NM C3471 light	Sheep	2952,5094	2,3,4	123.35	10
7	Karlby NM D4854b	Sheep	2952,5094	2,3,4,5	440.92	16
8	Karlby NM D4854c	Goat	3012,5094	2,3,4,5	283.37	6
9	Karlby NM D4854e	Sheep	2952,5094	3,4	156.27	2
12	Haraldskær NM C3705	Goat	3012,5094	3	167.78	11

Table S6. Statistics supporting the identification of the sheep/goat diagnostic collagen type-1 alpha-2 peptide [17,18].

Supplementary References

1. Cappellini E, Jensen LJ, Szklarczyk D, Ginolhac A, Fonseca RARd, et al. (2011) Proteomic Analysis of a Pleistocene Mammoth Femur Reveals More than One Hundred Ancient Bone Proteins. Journal of Proteome Research 11: 917–926.
2. Wiśniewski JR, Zougman A, Nagaraj N, Mann M (2009) Universal sample preparation method for proteome analysis. Nature Methods 6: 359-363.
3. Cappellini E, Gentry A, Palkopoulou E, Ishida Y, Cram D, et al. (2014) Resolution of the type material of the Asian Elephant, Elephas maximus Linnaeus, 1758 (Proboscidea, Elephantidae). Zoological Journal of the Linnean Society 170: 222-232.
4. Rappsilber J, Y YI, Mann M (2003) Stop and go extraction tips for matrix-assisted laser desorption/ionization, nanoelectrospray, and LC/MS sample pretreatment in proteomics. Analytical Chemestry 75: 663–670.
5. Kelstrup CD, Young C, Lavallee R, Nielsen ML, Olsen JV (2012) Optimized Fast and Sensitive Acquisition Methods for Shotgun Proteomics on a Quadrupole Orbitrap Mass Spectrometer. Journal of Proteome Research 11: 3487–3497.
6. Cox J, Mann M (2008) MaxQuant enables high peptide identification rates, individualized p.p.b.-range mass accuracies and proteome-wide protein quantification. Nature Biotechnology 26: 1367–1372.
7. Cox J, Neuhauser N, Michalski A, Scheltema RA, Olsen JV, et al. (2011) Andromeda: a peptide search engine integrated into the MaxQuant environment. . Journal of Proteome Research 10: 1794-1805.
8. Camacho C, Coulouris G, Avagyan V, Ma N, Papadopoulos J, et al. (2009) BLAST+: architecture and applications. BMC Bioinformatics 10 421.
9. Fatou JG (1978) Optical microscopy of fibres. Applied Fibre Science 1: 69-180.
10. Wildman AB (1954) The microscopy of animal textile fibres. Leeds: Wool Industries Research Association. 209 p.
11. Meyer M, Hülmann G, Seger H (2002) REM-Atlas zur Haarkutikulastruktur mitteleuropäischer Säugetiere. Hannover: Schaper. 248 p.
12. Hausman LA (1920) Structural characteristics of the hair of mammals. The American Naturalist 54: 96-523.
13. Luniak B (1953) The identification of textile fibres: qualitative and quantitative analysis of fibre blends. London: Sir Isaac Pitman & Sons, Ltd. 177 p.
14. Stoves JL (1957) Fibre microscopy: its technique and application. London: National Trade Press. 286 p.
15. Brunner H, Coman B (1974) The identification of Mammalian Hair. Melbourne: Inkata Press. 176 p.
16. Lochte T (1938) Atlas der menschlichen und tierischen Haare. Leipzig: Schöps. 306 p.
17. Buckley M, Kansa SW, Howard S, Campbell S, Thomas-Oates J, et al. (2010) Distinguishing between archaeological sheep and goat bones using a single collagen peptide. Journal of Archaeological Science 37: 13-20.
18. Campana MG, Robinson T, Campos PF, Tuross N (2013) Independent confirmation of a diagnostic sheep/goat peptide sequence through DNA analysis and further exploration of its taxonomic utility within the Bovidae. Journal of Archaeological Science 40: 1421-1424.

19. Solazzo C, Wadsley M, Dyer JM, Clerens S, Collins MJ, et al. (2013) Characterisation of novel α-keratin peptide markers for species identification in keratinous tissues using mass spectrometry. Rapid Communication in Mass Spectronomy 27: 1-14.
